# Lung adenocarcinoma harboring L858R and T790M mutations in epidermal growth factor receptor, with poor response to gefitinib: A case report

**DOI:** 10.3892/ol.2014.2321

**Published:** 2014-07-04

**Authors:** YUE FENG WANG, XIANHONG XIANG, XIAOJUAN PEI, SHUHUA LI, CUILAN TANG, LIANTANG WANG, ZUN-FU KE

**Affiliations:** 1 Department of Pathology, The First Affiliated Hospital of Sun Yat-sen University, Guangzhou, Guangdong 510080, P.R. China; 2 Department of Interventional Radiology, Huizhou Municipal Central Hospital, Huizhou, Guangdong 516001, P.R. China; 3 Department of Pathology, Huizhou Municipal Central Hospital, Huizhou, Guangdong 516001, P.R. China

**Keywords:** adenocarcinoma, biopsy, non-small cell lung cancer, epidermal growth factor receptor, mutation

## Abstract

Lung cancer is the leading cause of mortality among malignant diseases in humans worldwide. During the last decade, molecular targeted therapies for non-small cell lung cancer using first-generation, reversible epidermal growth factor receptor (EGFR) tyrosine kinase inhibitors (TKIs), including gefitinib, have been shown to be a promising approach for patients harboring activating mutations in EGFR. The current study reports a 77-year-old patient diagnosed with adenocarcinoma harboring L858R and T790M point mutations in the EGFR gene. The patient was treated with gefitinib as the second-line therapy, but no clinical benefit was observed. As the majority of patients with lung cancer receiving EGFR-TKI therapy acquire resistance, repeated biopsies and detection of the EGFR mutation state are beneficial for selecting appropriate treatments.

## Introduction

Lung cancer is the leading cause of cancer-related mortality worldwide (1). Recent studies on personalized treatment, conducted by selecting patients who are likely to respond to a particular therapeutic agent, may allow improved treatment efficacy. Activated mutations of the EGFR gene are normally located in exons 18 to 21, >90% of which consist of deletions in exon 19 and L858R substitution in exon 21 (2). Patients with non-small cell lung cancer (NSCLC) harboring mutations in the epidermal growth factor receptor (EGFR) gene exhibit a significant response to EGFR-tyrosine kinase inhibitors (TKIs) (3,4). Clinical trials have demonstrated that gefitinib improves progression-free and overall survival in the treatment of NSCLC (5). Gefitinib is now approved for these indications (6). Gefitinib has also been proposed for the treatment of patients with locally advanced or metastatic NSCLC with EGFR-activating mutations (7), which targets the tyrosine kinase (TK) domain of EGFR, inhibiting the downstream signaling processes for growth and proliferation. Mutations in the EGFR gene may also affect the behavior of the receptor and its response to inhibitors.

The majority of NSCLC patients with EGFR mutations initially benefit favorably from treatment with gefitinib, suggesting that these mutations promote tumorigenesis. However, among tumors that initially respond to EGFR-TKIs, the majority of patients eventually acquire resistance, often due to the emergence of a secondary mutation, such as T790M, in the kinase domain of EGFR (8). Patients with both L858R and T790M EGFR mutations are extremely rare (9). Written informed consent was obtained from the patient’s family.

## Case report

**Figure 1:**
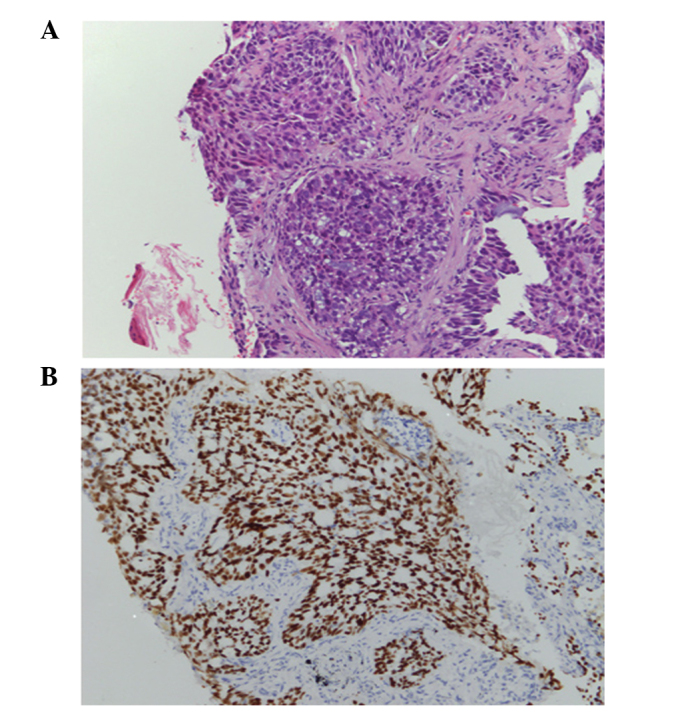
Histological morphology of the primary lung adenocarcinoma with a predominantly solid growth pattern. (A) Hematoxylin-eosin staining and (B) immunohistochemical staining for transcription factor-1 (magnification, ×10).

A 77-year-old male with a history of smoking was admitted to the Department of Interventional Radiology, The First Affiliated Hospital of Sun Yat-sen University (Guangzhou, China) in November 2011 due to an abnormal shadow in the right upper field following a chest X-ray. Physical examination revealed no significant abnormalities; however, computed tomography (CT) of the chest revealed a tumor measuring 66×74×80 mm in size in the right S1+2 with multiple lung and bone metastases (cT3N3M1; stage IV), according to the TNM classification (10). A transbronchial lung biopsy (TBLB) was conducted and the pathological diagnosis of the TBLB specimen was acinar adenocarcinoma (Fig. 1A). Immunohistochemical staining was positive for transcription factor-1 protein (Fig. 1B). Laboratory findings were within the normal range, with the exception of the carcinoembryonic antigen (CEA) level of 12.65 ng/ml (normal range, 0–3.4 ng/ml) in the serum. A diagnosis of lung adenocarcinoma was determined and the patient was treated with first-line chemotherapy consisting of cisplatin (80 mg/m^2^) and docetaxel (60 mg/m^2^), every three weeks for up to three cycles. However, no marked response was observed.

**Figure 2:**
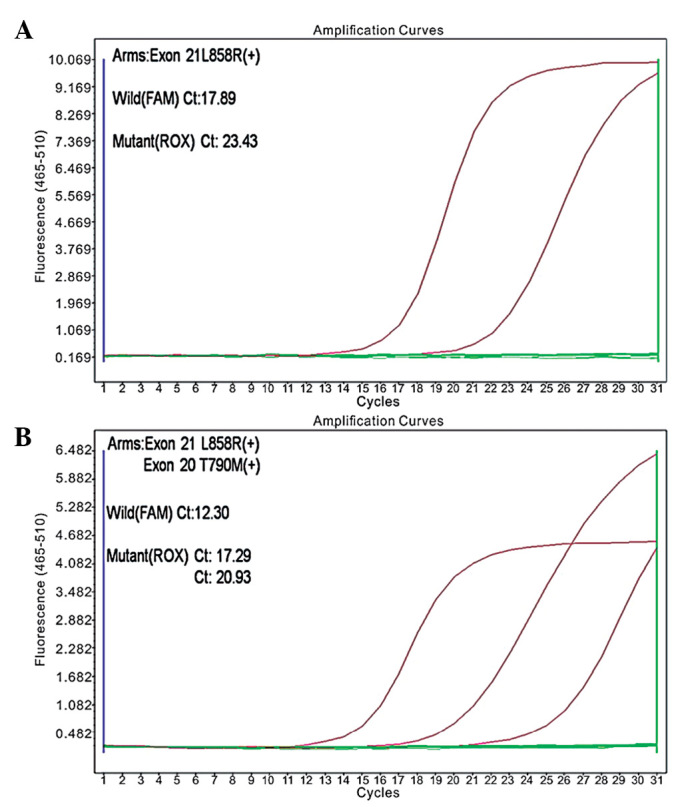
Epidermal growth factor receptor gene mutation was positive according to the amplification-refractory mutation system method. (A) L858R point mutation prior to the use of gefitinib, and (B) L858R and T790M point mutations following treatment with gefitinib.

**Figure 3:**
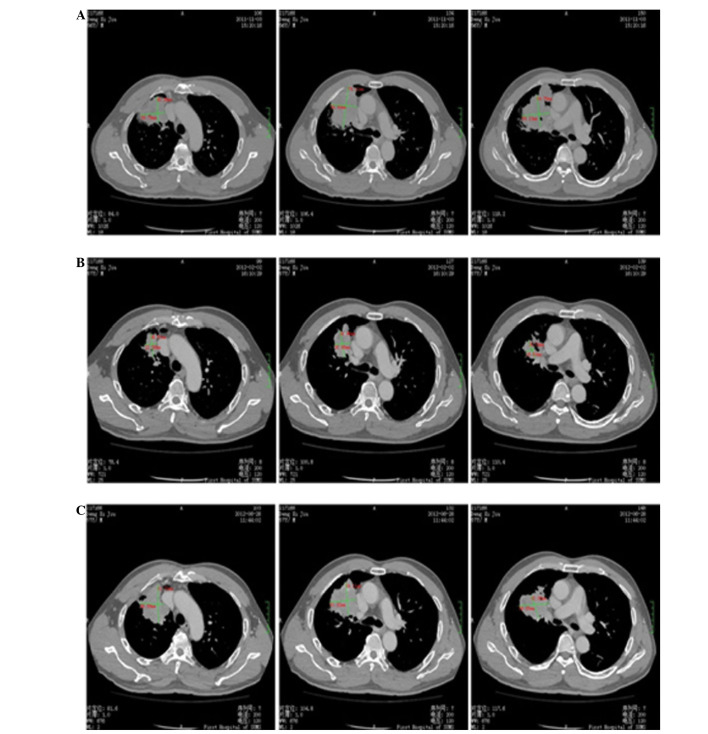
Computed tomography scans of the present non-small cell lung cancer patient. (A) Prior to treatment with gefitinib, (B) stable disease and (C) disease progression following long-term treatment with gefitinib.

Following the initial treatment, a mutation in the EGFR gene was identified (exon 21; L858R, in which the leucine at amino acid position 858 is replaced by arginine; Fig. 2A). The second-line chemotherapy was gefitinib (250 mg) once a day, administered between March and July 2012. The gefitinib therapy was effective, and no adverse events were reported. A CT scan of the thorax was performed in May 2012, which revealed residual disease in the right lung (51×72×51 cm in diameter) and few metastatic bone lesions (Fig. 3).

In November 2012, a further CT scan revealed a number of new lesions (one in the right lung, and several in the bone and brain), indicating disease progression. The patient was subjected to a rebiopsy to detect EGFR mutations, with analysis by the amplification refractory mutation system. L858R and T790M point mutations were detected in the tumor cells (Fig. 2B). Subsequently, the patient underwent three cycles of third-line chemotherapy (150 mg erlotinib per day for three months); however, further metastases emerged in the brain and, therefore, palliative care was administered in May 2013. The gefitinib therapy was discontinued to introduce the third-line chemotherapy, which induced an infusion reaction, and no remarkable response was observed. Following discontinuation of the third-line chemotherapy, the tumor growth induced empyema and the patient’s general condition gradually deteriorated and the patient succumbed to the disease in August, 2013.

## Discussion

It has been demonstrated that the majority of patients with lung cancer that are responsive to EGFR-TKIs harbor activating mutations in the TK domain of EGFR (11–13). This further supports the hypothesis that the identification of genetic signatures associated with oncogenic alterations may serve as predictive biomarkers for corresponding molecular target inhibitors. In comparison with smokers, EGFR mutations have consistently been found to be more common in non-smokers (14). In the present study, the patient did not have a history of smoking. Histopathologically, mutation rates among adenocarcinoma are predominantly higher than those in squamous cell lung carcinomas (15). The diagnosis of squamous cell carcinoma or adenocarcinoma is based on histomorphological grounds in cases where the appearances are characteristic; additionally, immunohistochemical staining is performed using antibodies against TTF-1, p63, M-CEA and CK. Immunohistochemical nuclear expression of TTF-1 also confirms a primary pulmonary origin. Furthermore, M-CEA-positive and p63-negative tumor cells indicate a glandular epithelium origin (16).

According to the Food and Drug Administration regulations, EGFR inhibitors have been approved as the first-line treatment for advanced NSCLC patients positive for EGFR-activating mutations (17). However, EGFR inhibitors are not used to treat patients with wild-type EGFR and, by contrast, a poor outcome has been observed in response to the treatment (18,19). Activating mutations of the EGFR gene are predominantly located in exons 18–21 and >90% are deletions in exon 19 or the L858R substitution in exon 21. These activating mutations are eligible for treatment with modern TKIs, for example gefitinib (20–22). Therefore, the accurate detection of EGFR mutations is critical for determining the efficacy in the adoption of gefitinib for advanced NSCLC in any given population. In the current study, the L858R point mutation of exon 21 was detected in the tumor cells, and an effective and curative outcome was observed following treatment with gefitinib. However, after several months, a CT scan revealed new lesions in the brain, indicating disease progression. The L858R point mutation of exon 21 and a compound T790M EGFR substitution mutation were detected in the tumor cells, which was consistent with the study by Pao *et al* (23), indicating that the efficacy of EGFR-TKIs in lung cancer is severely compromised by the rapid emergence of targeted therapy-resistant clones within one year.

In conclusion, the current study reports a rare case of lung cancer harboring an L858R point mutation of exon 21 and a compound T790M EGFR substitution mutation following treatment with gefitinib. However, following the detection of the T790M EGFR substitution mutation in the tumor cells, the patient exhibited poor curative effect when treatment with gefitinib was continued. Therefore, to improve the selection of optimal treatment regimens in individual patients, further investigation into determining the genetic causes of drug resistance at various points during the clinical course is required.
